# Supplementary material for: Mutations in PCYT2 disrupt etherlipid biosynthesis and cause a complex hereditary spastic paraplegia
Source: Brain. 2019 Oct 22;142(11):3382–97. doi: 10.1093/brain/awz291 (PMC6821184; doi:10.1093/brain/awz291)
Supplement: awz291_Supplementary_Data [file awz291_supplementary_data.zip › awz291-Suppl_data/Supplementary_Data4.zip!/Supplementary information 3 - fibroblast lipidomics.zip!/Fibroblast lipidomics/Controle vs Patient2/Statistical plots and data/PI species/Volcano plot_alternative.pdf]

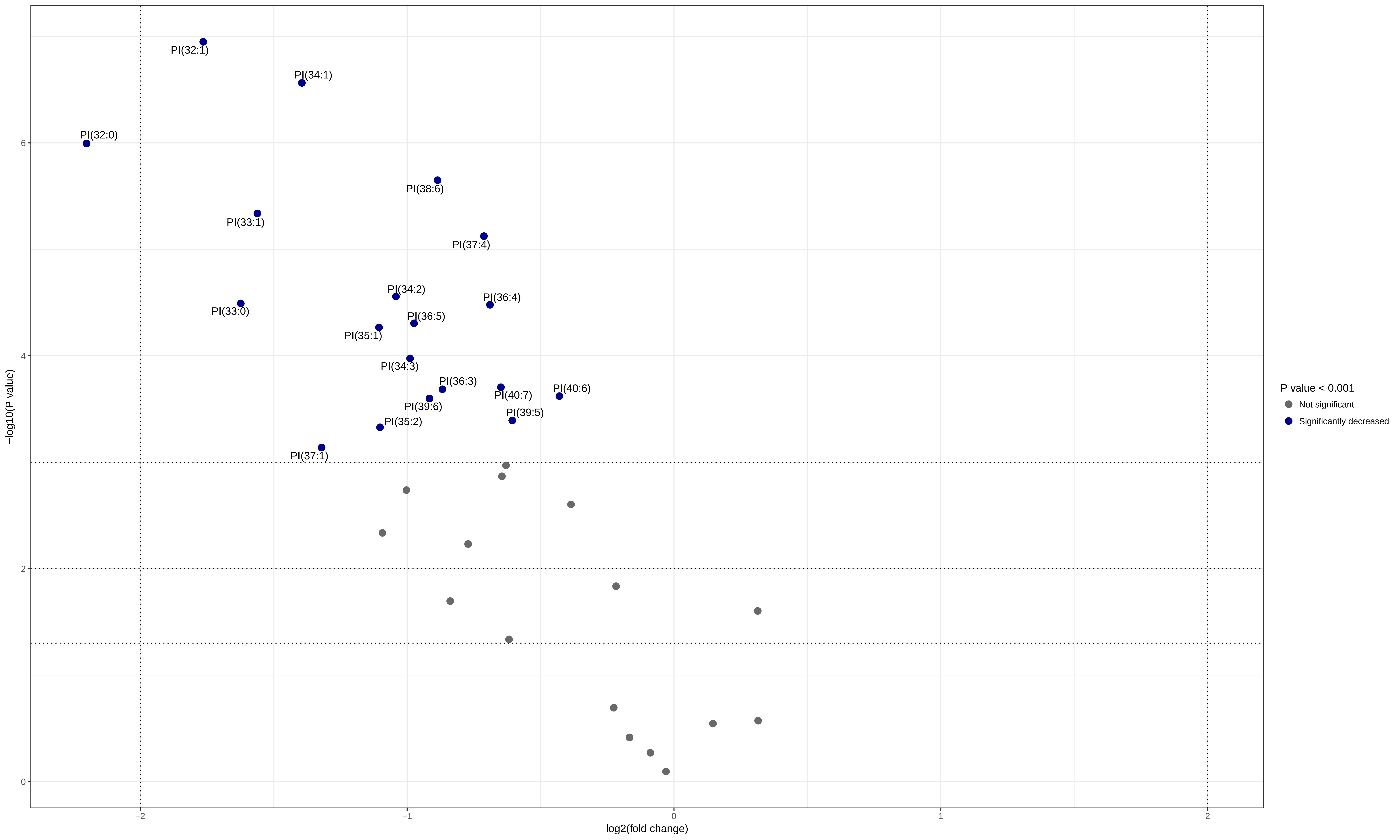

PI(32:1)
PI(34:1)
PI(32:0)
PI(38:6)
PI(33:1)
PI(37:4)
PI(34:2)
PI(36:4)
PI(33:0)
PI(36:5)
PI(35:1)
PI(34:3)
PI(36:3)
PI(40:6)
PI(40:7)
PI(39:6)
PI(39:5)
PI(35:2)
PI(37:1)
6
4
2
0
−2
−1
0
1
2
−log10(P value)
log2(fold change)
P value < 0.001
Not significant
Significantly decreased
